# Amperometric ion-selective nanoelectrodes for bioanalytical sensing and imaging

**DOI:** 10.1016/j.talanta.2026.129380

**Published:** 2026-01-09

**Authors:** Jiyeon Kim, Shigeru Amemiya

**Affiliations:** a Department of Chemistry, University of Rhode Island, Kingston, RI, 02881, United States; b Department of Chemistry, University of Pittsburgh, Pittsburgh, PA, 15260, United States

**Keywords:** Ion-selective nanoelectrode, Nanoscale scanning electrochemical, microscopy, In-vivo neuroanalysis, Ionophore, Bacteria, Nuclear pore complex

## Abstract

This review is focused on the recent applications of amperometric ion-selective nanoelectrodes as emerging electrochemical methods for bioanalytical sensing and imaging. The amperometric nanoelectrodes offer advantages over the potentiometric counterparts toward unprecedented in-vitro and in-vivo ion analysis of biological systems. The amperometric nanoelectrodes serve not only as electrochemical ion sensors based on highly selective ionophores but also as the tips of scanning electrochemical microscopy (SECM) to enable ion imaging with a spatial resolution of down to 30 nm. Moreover, the high biocompatibility and simple fabrication of ion-selective nanopipets and micropipets are attractive for in vivo bioanalysis. Specifically, we will introduce the principle of amperometric ion-selective nanoelectrodes as well as nanoscale SECM for bioanalytical sensing and imaging, respectively. Applications of amperometric ion-selective nanoelectrodes are exemplified by nanoscale SECM imaging of molecular transport at the single nuclear pore complex and chemical interactions among single bacterial cells. The powerful sensing applications of amperometric ion-selective nanoelectrodes are illustrated for the detection of carbonate generated by bacterial cells as well as both in-vitro and in-vivo detection of neurotransmitter acetylcholine.

## 1. Introduction

**Fig. 1.**
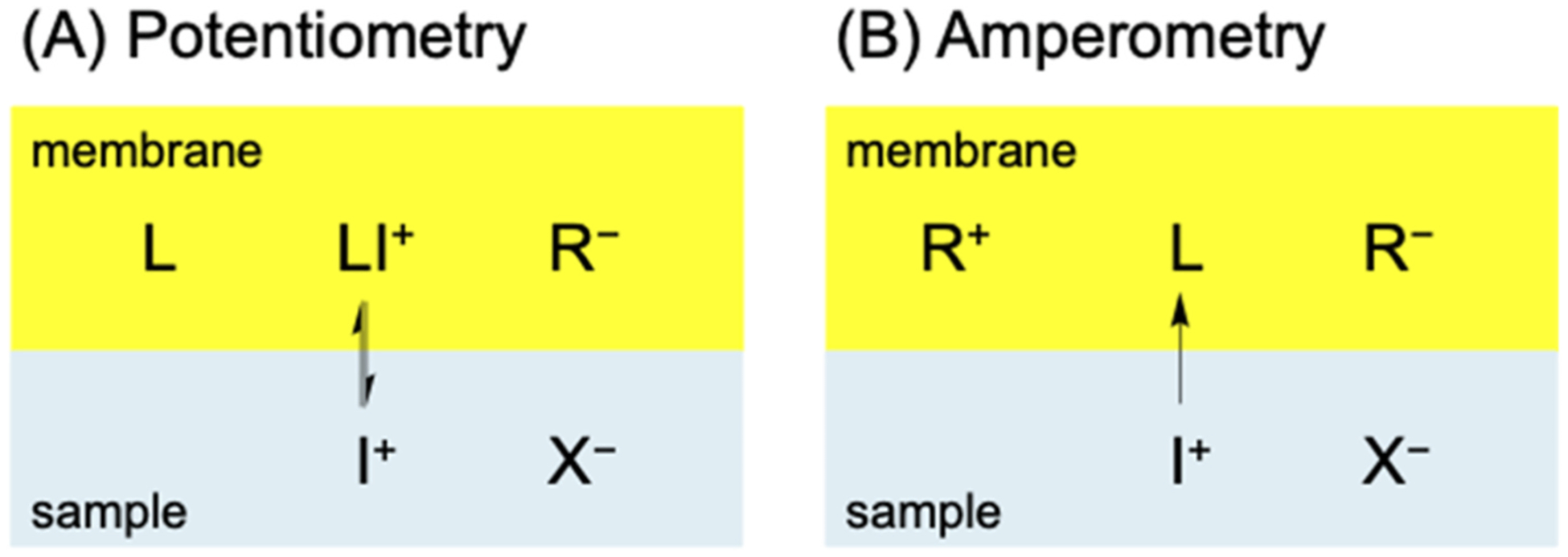
Initial compositions of (A) potentiometric and (B) amperometric ion-selective membranes in contact with an aqueous sample solution of a target analyte cation, I^+^. L, ionophore; LI^+^, ionophore–ion complex; R^−^, anionic site; R^+^, cationic site; X^−^, aqueous anion.

Amperometric ion-selective electrodes (ISEs) based on liquid or polymeric membranes [1–3] have emerged as advanced electrochemical sensors by employing the powerful ion-recognition chemistry of the traditional potentiometric counterparts [4,5]. In potentiometry (Fig. 1A), an analyte ion is present in both the aqueous sample and hydrophobic membrane to measure an equilibrium nernstian potential across the phase boundary. The resultant ion selectivity is controlled by the thermodynamics of ion transfer and ion–ionophore complexation. The potentiometric membrane is doped with an anionic (or cationic) site to accommodate an analyte cation (or anion) as an ionophore complex. By contrast, an amperometric membrane is doped with a pair of cationic and anionic sites as supporting electrolytes to polarize the phase boundary with the minimal membrane resistance (Fig. 1B). An analyte ion is initially present only in the sample and transferred into the membrane when an appropriate potential is externally applied across the phase boundary to measure the current response controlled by the ion-transfer rate. The resultant ion selectivity is determined not only by the same thermodynamics as potentiometry but also by the ion-transfer kinetics.

Potentiometric ISEs [4,5] have been developed for several decades not only to find a variety of important practical applications [6–8] but also to manifest the limitations of the equilibrium principle [4,9]. Potentiometric ISEs can detect more than 60 analyte ions [10–13] without the need for redox activity to enable clinical [14,15] and environmental [16,17] analysis and more recently wireless [18,19] and wearable [20,21] sensing [22]. An ionophore is added to a membrane to recognize an analyte ion with high selectivity, which is also required to reach a detection limit of down to low nanomolar [23]. An ion-selective membrane can be supported by a metal or carbon electrode with an ion-to-electron transducer to constitute all-solid-state ISEs [24,25]. Simple equilibrium potentiometry, however, limits the application of highly selective ion–ionophore recognition by allowing the detection of only one analyte ion with a single ISE and following the Nernst equation with lower sensitivity to an analyte ion with a higher charge [4,9].

Advantageously, the amperometric and voltammetric operation of liquid or polymeric membrane ISEs can overcome the limitations of the potentiometric counterpart in selectivity, sensitivity, and detection limit. Traditionally, a bulk organic electrolyte was employed as an ion-selective phase for amperometric and voltammetric ISEs to require an unconventional four-electrode cell and a large amount of ionophores [26]. These practical limitations can be overcome by operating solid-state ISEs in amperometric and voltammetric modes [27,28]. The current response varies with the charge of an analyte ion to yield high sensitivity to polyions [27,29]. Moreover, ion selectivity can be controlled voltammetrically to detect multiple ions at different potentials by using a single ISE [30,31]. Furthermore, an analyte ion can be preconcentrated into the thin membrane and then transferred reversely by stripping voltammetry to achieve a detection limit of down to picomolars [27,32–36]. The low detection limits can be achieved by eliminating adventitious contaminant ions, which were detectable voltammetrically but not potentiometrically to unknowingly compromise the detection limits at nanomolar levels.

This article features the recent advancement of amperometric ion-selective nanoelectrodes as a powerful alternative to the potentiometric counterparts [37] for bioanalytical sensing and imaging. Potentiometric ISEs have been miniaturized to the nanometer scale and applied for the in-vitro and in-vivo analysis of physiological ions for many years [38]. The synergy between the high biocompatibility and powerful amperometric operation of nanoscale ISEs has enabled unprecedented bioanalytical applications of the emerging electrochemical method [39]. Specifically, we will introduce the principle, fabrication, and characterization of amperometric ion-selective nanoelectrodes based on glass or quartz nanopipets. Amperometric ion-selective nanoelectrodes can be used as a tip of nanoscale scanning electrochemical microscopy [40,41] for high-resolution imaging of biological events as represented by molecular transport through single nuclear pore complexes [42] and chemical interactions among single bacterial cells [43,44]. Advantageously, an amperometric response is sensitive to the tip–substrate distance in contrast to the potentiometric counterpart, thereby facilitating the precise distance control required for high-resolution SECM measurements [45]. Moreover, the highly selective ionohores developed for potentiometric ISEs are compatible with amperometric nanoelectrodes as exemplified for the detection of carbonante in bacterial suspensions [46]. Furthermore, ionophore-based amperometric nanoelectrodes [47,48] and microelectrodes [49,50] enabled the in vitro and in vivo detection of acetylcholine, respectively, as an imperative neurotransmitter without redox activity.

Fundamentally, amperometric ion-selective nanoelectrodes are built upon electrochemistry at liquid/liquid interfaces also known as the interface between two immiscible electrolyte solutions (ITIES) [1–3]. This important topic of electrochemistry has been introduced and discussed in a number of seminal [26,51,52] and recent [2,53] reviews, which are complementary to this review. Both thermodynamics and kinetics of ion transfer across liquid/liquid interfaces have been well established theoretically [54] and verified experimentally even at the nanoscale [46,55–58]. Heterogeneous electron transfer across the liquid/liquid interfaces can be described within the framework of Marcus theory [59–61] and examined experimentally by using SECM [62]. The structure and thickness of liquid/liquid interfaces have also been assessed both theoretically and experimentally [63]. Practically, electrochemistry at liquid/liquid interfaces has been useful for various interdisciplinary areas of nanoscience and nanotechnology including ion sensing at an array of nanoscale liquid/liquid interfaces [64], interfacial assembly and characterization of nanomaterials [65], and electrochemical detection of single nanoparticles [66,67] including nanoemulsions as nanoscale liquid/liquid interfaces [68–71]. Furthermore, nanoscale liquid/liquid interfaces are naturally formed by phase separation in biological cells [72] and can be studied or relevant electrochemically [73].

## 2. Amperometric ion-selective nanoelectrode

**Fig. 2.**
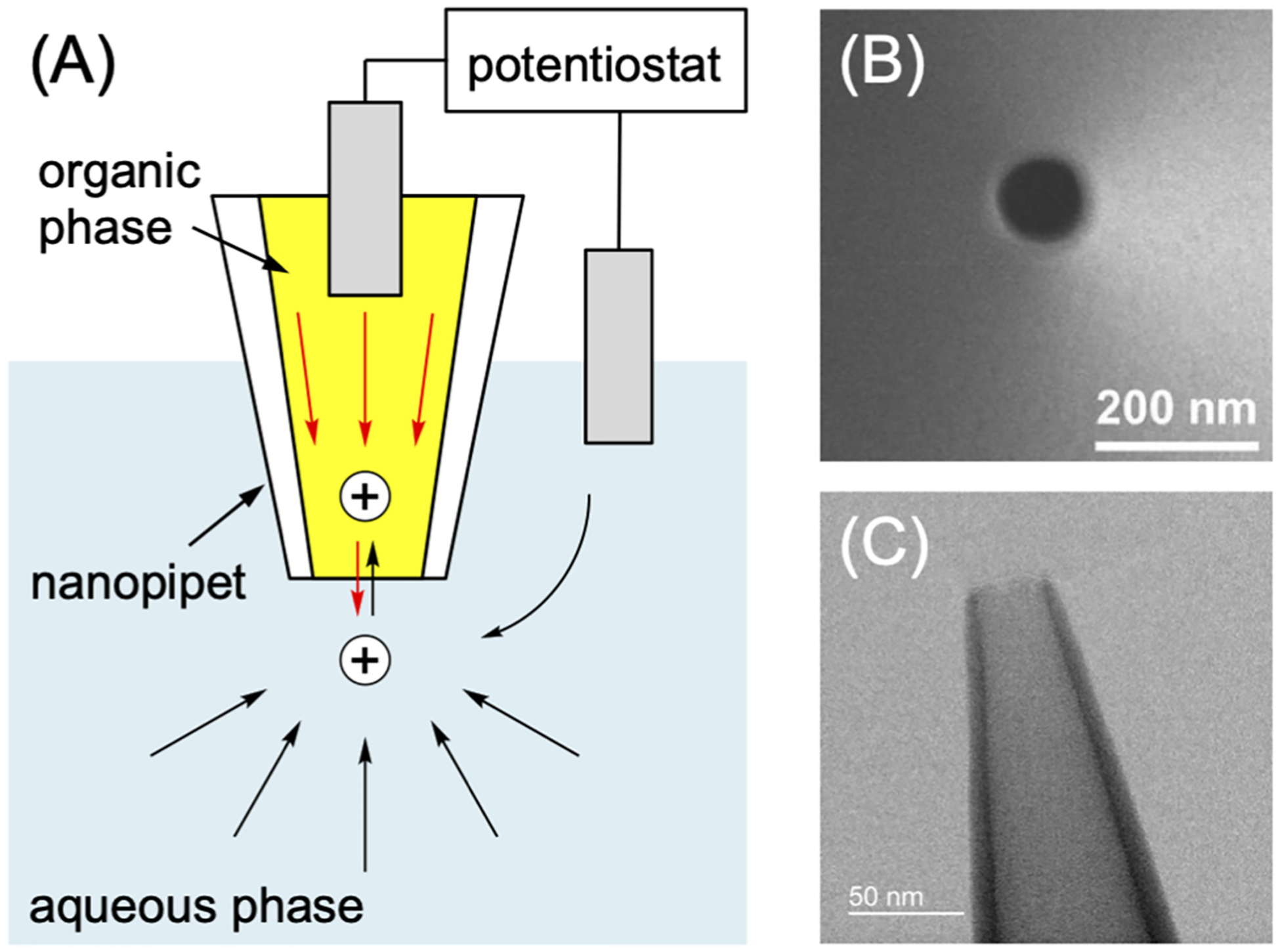
(A) Amperometric ion-selective nanoelectrode based on ion transfer across the liquid/liquid interface supported by a nanopipet. Reproduced from Ref. [1] with permission from Elsevier. (B) SEM image of a quartz nanopipet coated with a thin Au film. Reproduced from Ref. [75] with permission from the American Chemical Society. (C) TEM image of an as-pulled quartz nanopipet. Reproduced from Ref. [76] with permission from the American Chemical Society.

A glass or quartz nanopipet is filled with a water-immiscible organic solution of highly lipophilic electrolytes and immersed in an aqueous electrolyte solution to serve as an amperometric ion-selective nanoelectrode [2] (Fig. 2A). The organic phase may contain only hydrophobic electrolytes to drive the simple transfer of an analyte ion across the nanoscale liquid/liquid interface

(1) $${\text{I}}^{\text{z}}(\text{aq})⇌{\text{I}}^{\text{z}}(\text{org})$$

where ${\text{I}}^{\text{z}}$ is an analyte ion with a charge of z in the aqueous or organic phase. The mechanism of simple ion transfer has been described by Marcus theory [54], which is based on the water fingers predicted by molecular dynamics simulation [74] and was assessed voltammetrically by using ion-selective nanoelectrodes [55,56]. Simple ion transfer may be facilitated by an ionophore, L, in the organic phase through the non-concerted mechanism

(2) $${\text{I}}^{\text{z}}(\text{org})+\text{L}(\text{org})⇌{\text{LI}}^{\text{z}}\;(\text{org})$$

where ${\text{LI}}^{\text{z}}$ is an ionophore–ion complex. Alternatively, facilitated ion transfer may be driven through the concerted mechanism

(3) $${\text{I}}^{\text{z}}(\text{aq})+\text{L}(\text{org})⇌{\text{LI}}^{\text{z}}\;(\text{org})$$

Both mechanisms are equivalent thermodynamically as represented by the identical formal potential but not equivalent kinetically [77] and can be discriminated from each other electrochemically as demonstrated by using ion-selective micropipets [57] and nanopipets [46,58].

Experimentally, a metal electrode is inserted into the organic phase to externally control the potential across the nanoscale liquid/liquid interface against an aqueous reference electrode and measure the current based on interfacial ion transfer. The interfacial potential must be more negative (or positive) with respect to the formal potential of cation (or anion) transfer to accelerate the ion-transfer rate. The interfacial potential can be controlled accurately without a substantial Ohmic potential drop in the organic phase, which contains a high concentration of electrolytes and carries only low current. With sufficient potentials, the resultant ionic current can be limited by the steady-state diffusion of the transferred ions from the aqueous solution to the interface as given by

(4) $$i_{\text{T},\infty }=4\text{xzFD}c_{0}a$$

where x is a function of RG [78] ($=r_{\text{g}}/a\approx 1.4$ typically; a and $r_{\text{g}}$ are the inner and outer radii of a pipet tip), F is the Faraday constant, and D and $c_{0}$ are the diffusion coefficient and concentration of the transferred ions in the bulk aqueous solution. Eq (4) is valid for facilitated ion transfer when an excess amount of an ionophore is present in the organic phase to deplete an analyte ion near the interface [79]. The transferred ions efficiently diffuse away into the organic phase through the tapered region of the nanopipet to yield a steady-state cyclic voltammogram. By contrast, a reverse peak current is expected at the micropipet-supported interface [79].

An experimental protocol has been well established and detailed [46] to obtain the reproducible current response of ion-selective electrodes based on the organic-filled nanopipets. A glass or quartz capillary is heat-pulled by using a computer-controlled CO_2_-laser puller to reproducibly obtain a nanopipet with a tip diameter of down to 10 nm [80]. The tip size can be imaged by scanning electron microscopy (SEM) of a nanopipet coated with a ~3 nm-thick metal layer (Fig. 2B [75]) or directly by transmission electron microscopy (TEM; Fig. 2C [76]). In TEM, the electron-beam intensity must be optimized to prevent the melting of the insulating nanopipet tip. The size of the nanoscale liquid/liquid interface is determined electrochemically from the diffusion-limited steady-state current (eq (4)) or more strictly from the approach curve of SECM. The liquid/liquid interface is spontaneously and reproducibly formed at the very tip of a nanopipet to minimize the contact area between the mutually immiscible solutions when the inner wall of the organic-filled pipet is tapered and sufficiently hydrophobic. The wall of a nanopipet can be reproducibly rendered hydrophobic by reacting under a dry atmosphere with N,N-dimethyltrimethylsilylamine as a silanization reagent with moderate reactivity [81]. The tip end can be blocked by a polymeric material when the tip is treated with a highly reactive silanization reagent, e.g., trimethylchlorosilane, under a wet atmosphere [55]. Reproducible current responses are obtained by preventing the adventitious contamination of a silanized nanopipet in a clean hood when the pipet is filled with an organic electrolyte solution, equipped with a clean and corrosion-resistant Cu/Ni wire, and inspected by light microscopy [46].

## 3. Nanoscale scanning electrochemical microscopy (SECM)

**Fig. 3.**
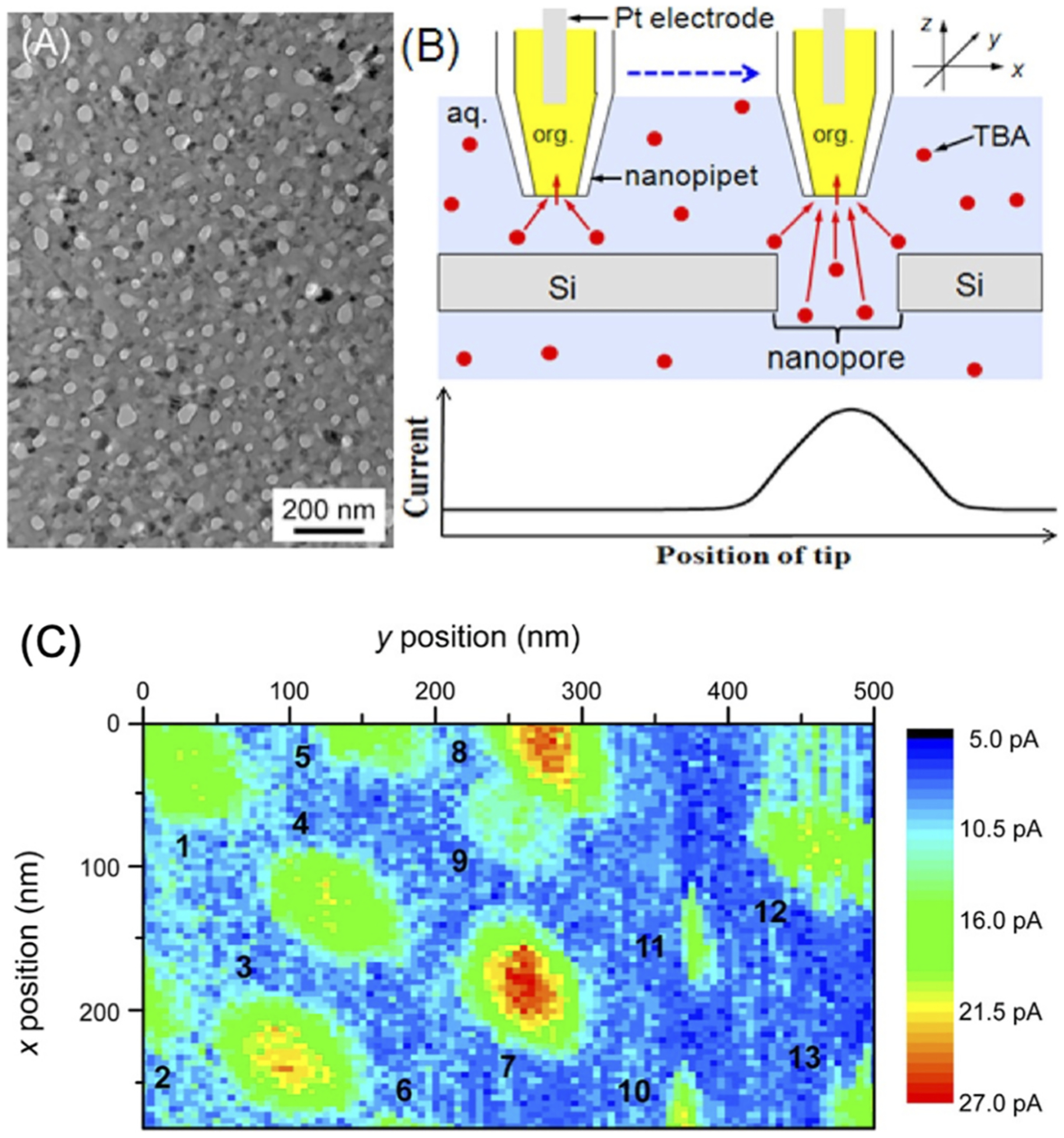
(A) TEM image of a nanoporous Si membrane, (B) SECM imaging of the membrane by an amperometric ion-selective nanopipet with a 30 nm diameter, and (C) the resultant image of thirteen nanopores in 10 mM TBACl and 0.3 M KCl. The nanopipet was filled with the 1,2-dichloroethane solution of tetradodecylammonium (TDDA) tetrakis (pentafluorophenyl)borate (TFAB). Reproduced from Ref. [82] with permission from the American Chemical Society.

Amperometric ion-selective nanoelectrodes were employed as tips of nanoscale SECM [40,41] to achieve a spatial resolution of less than 100 nm for imaging a substrate fully immersed in the solution for the first time [82]. By contrast, an unconventional setup was used to achieve a higher spatial resolution of ~1 nm for SECM imaging of biological macromolecules, which were placed on a mica substrate covered with a thin layer of condensed water (a few nanometers or less) [83]. Specifically, reproducible nanoscale SECM imaging was enabled by the precise positioning of an amperometric nanoelectrode tip to image ion transport through single solid-state nanopores with a spatial resolution of 30 nm [76,82] (Fig. 3A). An ~30 nm-diameter ion-selective nanopipet was moved vertically by monitoring the amperometric tip current response to tetrabutylammonium (TBA^+^). The tip current was lowered as the tip was positioned near the insulating part of the membrane, which hindered the diffusion of TBA^+^ to the tip (Fig. 3B). The tip current was peaked over a nanopore as the tip was scanned laterally over a nanoporous membrane by maintaining a short tip–substrate distance of ~1 nm. The precise control of the tip position was enabled by a nanoscale SECM instrument equipped with an isothermal chamber and closed-loop piezo actuators based on capacitive feedback [84,85]. The finite element analysis of SECM images confirmed that the high spatial resolution of ~30 nm was limited by the tip diameter of a nanopipet. More quantitatively, the pore diameter in the SECM image was larger than the actual pore diameter owing to the lateral diffusion of TBA^+^ in the gap between the tip and the pore. The diffusion effect on the pore size in the SECM image was ensured by employing a periodic array of solid-state nanopores with uniform pore diameters of 100 nm as determined by TEM [76]. In addition, the tip diameter of a nanopipet was determined by TEM (Fig. 1C) to ensure the reliability of quantitative finite element analysis.

The application of amperometric ion-selective nanopipets for SECM imaging of single nanopore permeability is significant. The nanoporous membranes are molecularly thin (~10 nm in thickness) to yield much higher permeability than traditional nanoporous membranes. The ultrathin membranes are robust enough to self-stand in the solution [86], thereby serving as superior alternatives for hemodialysis [87] and cell co-culture [88] as well as enabling nanomaterials for wearable artificial kidney [89] and tissue-on-chips [90]. Moreover, the ultrathin nanoporous membranes can be the solid-state morphological mimic of the nuclear envelope perforated by nuclear pore complexes with a pore radius of 24 nm and a pore length of 35 nm [91].

An amperometric ion-selective nanoelectrode offers important advantages for high-resolution SECM imaging of biological samples. An ion-selective nanoelectrode is robust in comparison with a solid nanoelectrode based on an electronic conductor, e.g., platinum [92] and carbon [93], which is readily damaged by electrostatic charge. Electrostatic charges may be injected from an operator to the contact wire of a nanoelectrode or the operational amplifier of a potentiostat. The respective sources of electrostatic charges can be eliminated by grounding the operator under high humidity and by maintaining the connection between the potentiostat and the electrochemical cell [84, 92]. The absence of tip damage can be confirmed by SEM or TEM after the use of the nanotip [84]. In addition, the use of an ion-transfer reaction at the tip and the substrate is advantageous for high-resolution SECM imaging. When an electron-transfer reaction is employed, an electron may tunnel across the solution between the nanotip and the substrate to operate in the mode of scanning tunneling microscopy. Without special care [94], it is hard to distinguish between the tunneling current and the faradaic current at the tip, thereby leaving ambiguity in the interpretation of the resultant image [95].

## 4. Molecular transport through the nuclear pore complex (NPC)

Recently, we employed amperometric ion-selective nanoppipets as SECM tips to image TBA^+^ transport through single NPCs with a spatial resolution of ~30 nm, which was high enough to distinguish between plugged and unplugged NPCs [42]. The NPC solely transports small molecules, proteins, and RNAs between the nucleus and cytoplasm of a eukaryotic cell to play imperative biological [96,97] and biomedical [98,99] roles. The NPC is crucial to the regulation of gene expression [100,101] and is linked to many diseases [102,103] including cancers [104], neuronal diseases [105,106], and infectious diseases [107,108]. Single NPC imaging represents the unprecedented power and significance of nanoscale SECM, which determined the impermeability of plugged NPCs to support the hypothesis that the central plugs are not permeable transporters intrinsic to the NPC [109] but are impermeable cargos, e.g., ribonucleoproteins (RNPs), captured during translocation through the central pathway [110]. These two origins have been debated for decades [109–111] but cannot be distinguished by structural imaging of plugged NPCs using cryo-electron tomography [111–116] or AFM [117,118].

**Fig. 4.**
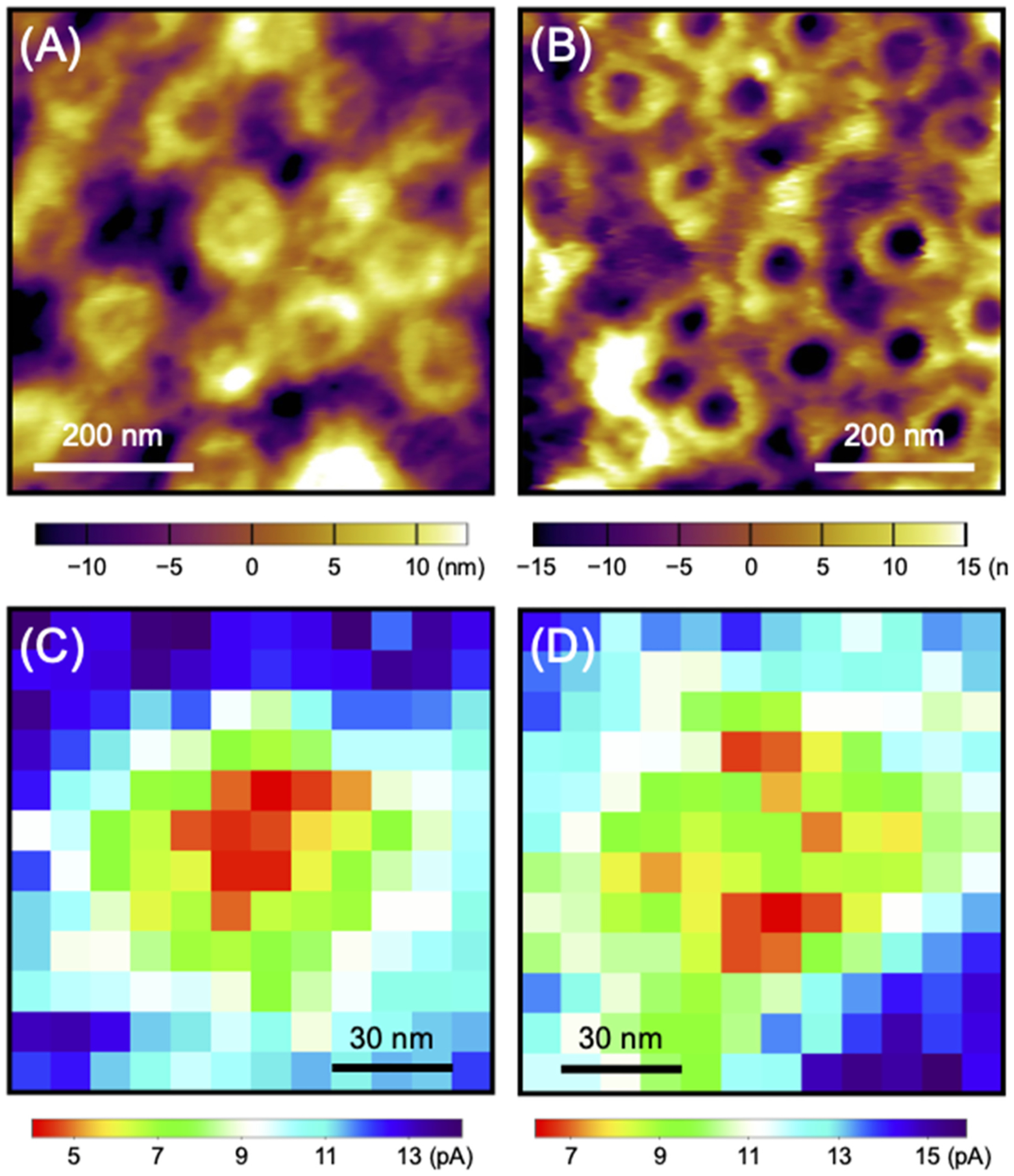
(A) and (B) AFM images of multiple NPCs and (C) and (D) amperometric SECM images of TBA^+^ transport through single NPCs with ~30-nm diameter ion-selective nanopipets. NPCs were plugged in (A) and (C) and unplugged in (B) and (D). The nanopipet was filled with the 1,2-dichloroethane solution of TDDATFAB. Reproduced from Ref. [42] with permission from the American Chemical Society.

We employed AFM to image the cytoplasmic side of plugged and unplugged NPCs (Fig. 4A and B, respectively) at the NE isolated from the large nucleus of a *Xenopus laevis* oocyte and incubated in buffer solutions containing physiological sub-micromolar or low millimolar concentrations of Ca^2+^, respectively [119]. The NE was fixed with glutaraldehyde to maintain the plugged and unplugged states of the NPCs [118] without altering the permeability to a small probe ion, TBA^+^, as confirmed by microscale SECM. We employed ~30 nm-diameter ion-selective pipets to distinguish between plugged and unplugged NPCs by SECM imaging. The tip current response to TBA^+^ stayed low over the center of a plugged NPC (Fig. 4C) but increased detectably over the center of an unplugged NPC (Fig. 4D). The size of the plug is equivalent to that of RNP [110] and is large enough to detectably prevent the flux of TBA^+^ driven through the NPC by the nanopipet. The central location of the impermeable plug supports our hypothesis that the NPC is divided into the central pathway for RNA/RNP export [42] and the peripheral pathway for protein import [120,121]. Technologically, the detection of the macromolecular plug is a crucial step toward SECM-based Coulter counting [122,123] to resolve the translocation of single macromolecules through the single NPC.

It should be noted that the limitation of nanoscale electrodes is the difficulty in performing transient measurements, which are feasible [124]. Alternatively, amperometric ion-selective microelectrodes were employed for the transient SECM studies of interactions between NPCs and arginine-containing dipeptide repeats (DPRs) [125,126] as NPC-blocking neurotoxines [127]. Arginine-containing DPRs of proteins resulting from the disordered *C9orf72* gene have been suspected as a potential cause of serious neurological diseases such as amyotrophic lateral sclerosis and frontotemporal dementia, also known as Lou Gehrig’s and Pick’s diseases, respectively [128]. Experimentally, an ion-selective microelectrode was doped with dinonylnaphthalene sulfonate as a negatively charged ionophore for polycations [129,130] to measure a chronoamperometric response to twenty repeats of neurotoxic glycine–arginine, GR_20_, and proline–arginine, PR_20_, as well as protamine [131,132]. The tip was positioned near the nuclear envelope of the *Xenopus* oocyte nucleus supported by a microporous membrane [119] and equilibrated with DPRs in the aqueous solution. The equilibrium was disturbed instantaneously by stepping the tip potential to amperometrically transfer the polycationic DPRs from the aqueous solution [131,132]. The tip current was enhanced by the desorption of DPRs from the NPCs and analyzed numerically to demonstrate that the DPRs are bound to the NPC nanopore for much longer than the physiological nuclear transport receptors, thereby clogging the nanopore to express neurotoxicity.

## 5. Chemical interactions among bacteria

**Fig. 5.**
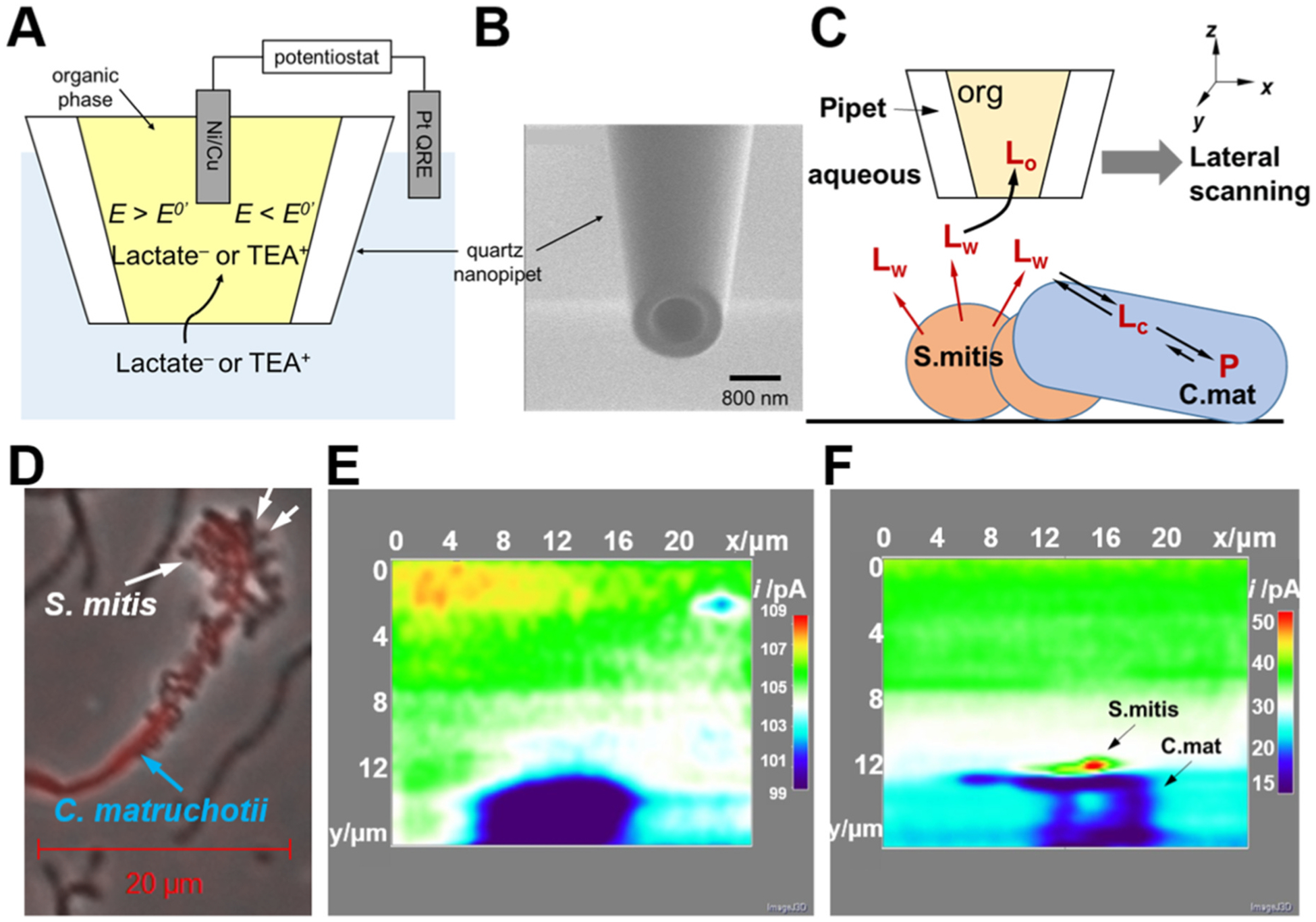
(A) Scheme of a submicropipet-supported ITIES to directly probe TEA^+^ transfer or lactate transfer. (B) SEM image of a pipet tip, (C) Scheme of constant-height SECM imaging (lateral scanning along x and y axis) over *S. mitis* and *C. matruchotii* coculture during *in situ* production/consumption of lactate, respectively, (D) Optical microscopic image of *C. matruchotii* (blue arrow) and *S. mitis* (white arrows) coculture. The nanopipet was filled with the 1,2-dichloroethane solution of 0.1 M TDDATFAB. Constant-height SECM images based on (E) TEA^+^ transfer and (F) lactate transfer, tip scan rate at 100 nm/100 ms. Reproduced from Ref. [43] with permission from the American Chemical Society.

In this section, we introduce the application of amperometric ion-selective nanpipets for the detection of lactate [43], ${\text{CO}}_{3}^{2-}$ [46], and antimicrobial drugs [75] to understand chemical interactions among bacteria. Real-time sensing and monitoring of the interaction between bacteria and molecules, e.g., exogenous chemicals or metabolites, are critical to chemically understand bacterial physiology especially for clinical and energy applications. We recently employed nanoscale SECM with ion-selective nanopipets to address the real-time commensal metabolic interaction in the oral microbiome [43,44]. The human oral microbiome heavily influences the status of not only oral but also systemic disease development through different microbial compositions and complex signaling between microbes [133]. Despite the recent discovery of the unique biogeography among oral microbes by optical microscopy [134,135], it has not been well understood how stable communities are maintained and how they may preserve health with ex-situ and ensemble measurements. Direct probing of metabolites in real-time near single bacterial cells at high spatial resolution is highly demanded to elucidate the metabolic interaction between oral microbes, and to mechanistically understand bacterial performance. We investigated the coculture of two highly abundant species in the human supragingival plaque, *Streptococcus mitis* (*S. mitis*) and *Corynebacterium matruchotii* (*C. matruchotii*) by applying nanoscale SECM and a submicropipet-supported ITIES as an SECM probe tip not only to image the permeability of *S. mitis* and *C. matruchotii* membranes to tetraethylammonium (TEA^+^) probe for a topographic map but also to real-time visualize the metabolic interaction between these microbes via lactate production/consumption at a single cell level (Fig. 5).

The tip current response to TEA^+^ transfer decreased detectably over bacteria due to the hindered diffusion of TEA^+^ to a pipet tip, where a lump appeared without discrimination of the two microbes because of their similar height and similar membrane permeability in the topographical SECM image (Fig. 5E). Dramatic contrast was observed with tip current responses to lactate transfer in the same scanned area, where the tip current over a spherical *S. mitis* increased c.a., 167 % of diffusion-limited steady-state current, $i_{\text{T},\infty }$, whereas the tip current over a long filamentous *C. matruchotii* decreased by up to 47 % of $i_{\text{T},\infty }$ (Fig. 5F). High tip currents above *S. mitis* are mainly due to the steady excretion of lactate as a fermentation product. Meanwhile, low tip currents above *C. matruchotii* are attributed to swift *in situ* consumption of lactate by *C. matruchotii*, where the competitive consumption of lactate by a pipet tip and *C. matruchotii* leads to lower tip currents than currents from the hindered diffusional flux of lactate by the *C. matruchotii* bacterial membrane. Notably, nanoscale SECM with an ion-selective pipet tip visualized real-time chemical exchanges between *S. mitis* and *C. matruchotii* through simultaneous lactate production/consumption, thereby for the first time proving their commensal relationship at a single cell level. Quantitative assessment of currents in SECM images enabled to determine the passive permeability of both bacterial membranes to the free diffusion of TEA^+^, the lactate concentration and its production rate by a single *S. mitis*, and a lactate oxidation rate by an individual *C. matruchotii*, thereby demonstrating a mechanism of *in situ* metabolic interaction between oral commensals at the single cell level and supporting the observed in vivo spatial arrangements of these microbes.

**Fig. 6.**
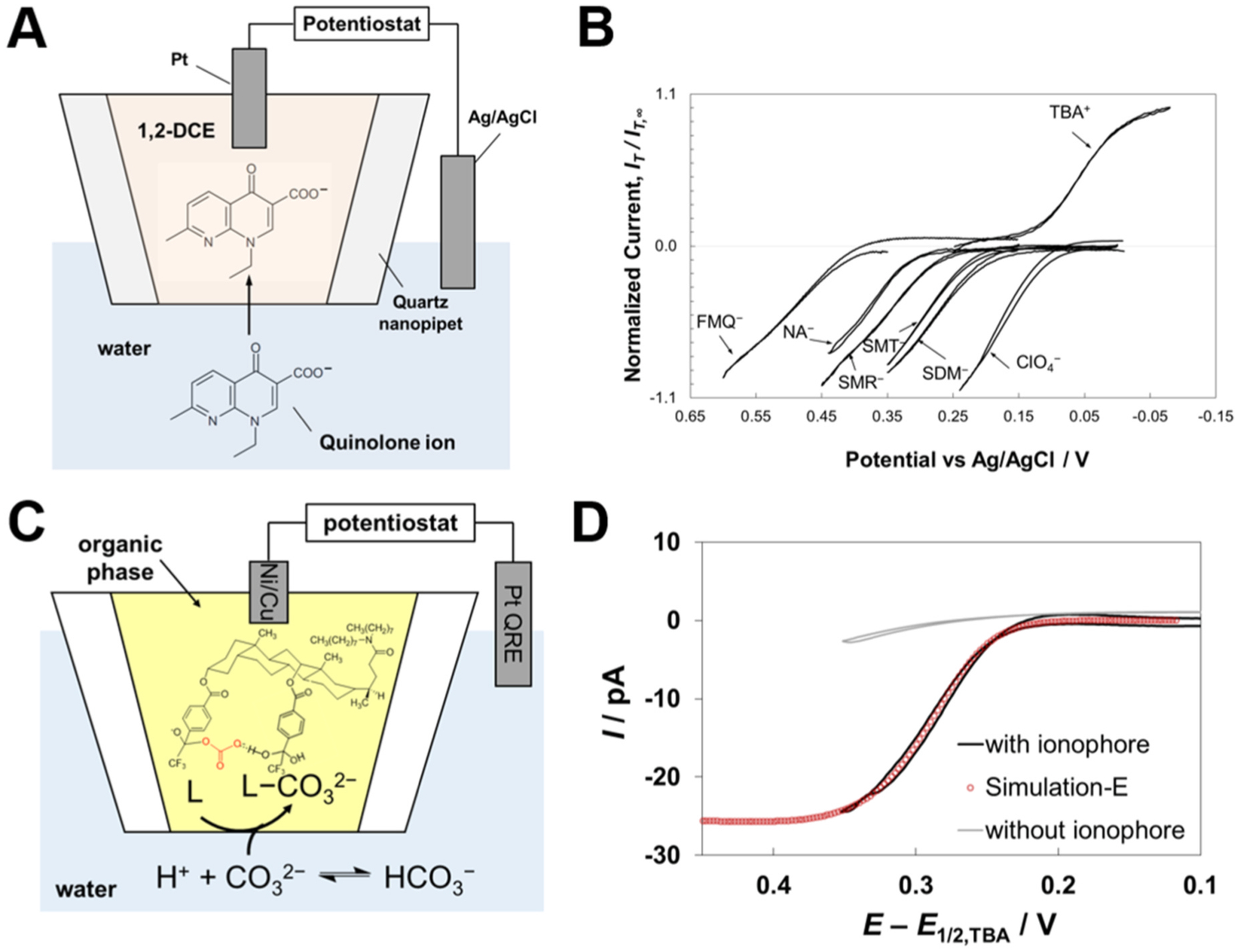
(A) Scheme of drug-ion transfer at a water/1,2-DCE interface measured by nanopipet voltammetry. (B) Normalized voltammograms of various drug ions, TBA^+^, and ${\text{ClO}}_{4}^{-}$ transferred across a water/1,2-DCE interface supported at a nanopipet. The nanopipet was filled with the 1,2-dichloroethane solution of 0.1 M TDDATFAB. Parts (A) and (B) were reproduced from Ref. [75] with permission from the American Chemical Society. (C) Scheme of facilitated ${\text{CO}}_{3}^{2-}$ transfer at a water/1,2-DCE interface measured by nanopipet voltammetry. (D) Steady-state voltammograms with and without ${\text{CO}}_{3}^{2-}$ ionophore VII for facilitated ${\text{CO}}_{3}^{2-}$ transfer across the 1,2-DCE/water interface obtained with a nanopipet filled with the solution of *premade* 30 mM ionophore and 0.1 M TDDATFAB. A simulated voltammogram is based on the E mechanism. Parts (C) and (D) were reproduced from Ref. [46] with permission from the American Chemical Society.

Also, we applied steady-state voltammetry using nanopipet-supported ITIES to directly sense pristine antimicrobial drug-ions under physiological conditions, and quantitatively study their transport-kinetics at the water/oil interface [75]. Effective delivery and accumulation of antimicrobial agents into the microbial organism are essential for the treatment of bacterial infections [136]. The transport of hydrophilic drug molecules often encounters a robust barrier of the hydrophobic double membrane cell envelope, thus leading to drug-resistance in Gram-negative bacteria [137]. Accordingly, a deeper understanding of the transport of charged molecules through the bacterial membrane is needed to remediate the antibacterial resistance. ITIES can serve as an artificial cellular membrane system to provide insightful surrogates for kinetic study of drug entry through bacterial cytoplasmic membranes. Markedly slow kinetics of drug-ion transfer with quinolones and sulfonamides were observed by nanopipet voltammetry, where drug-ion transfer is ~3 orders of magnitude slower than TBA transfer [75]. Further, the effective hydrophilicity of drug ions was quantitatively estimated from half-wave potentials of the obtained voltammograms compared to ${\text{ClO}}_{4}^{-}$ [138]

(5) $$\frac{P_{i}}{P_{{\text{ClO}}_{4}^{-}}}=\text{exp}\left[-\frac{zF\left(E_{\text{i}}-E_{{\text{ClO}}_{4}^{-}}\right)}{RT}\right]$$

where $P_{\text{i}}$ and $P_{{\text{ClO}}_{4}^{-}}$ are partition coefficients of drug ion, i and ${\text{ClO}}_{4}^{-}$ between the aqueous buffer and 1,2-dichloroethane (1,2-DCE) phases, z is a net charge (z=1) of the ions, and $E_{\text{i}}$ and $E_{{\text{ClO}}_{4}^{-}}$ are the half-wave potentials of the corresponding ions as determined from experimental voltammograms (Fig. 6B [75]).

Remarkably, the effective hydrophilicity of drug ions was 2–6 orders of magnitude higher than ${\text{ClO}}_{4}^{-}$, exclusively attributed to strong interaction between water molecules and localized negative charges on carboxylate or amide group of deprotonated quinolone or sulfonamide during interfacial IT, thereby increasing energy barriers upon the water-finger formation and breaking with drug ions near the interface [139] and leading to sluggish kinetics. By employing nanoscale SECM and ion-selective pipets as SECM probes, real-time drug permeation will be monitored at a single bacterial cell to elucidate the relationship between pristine drug structures and their membrane permeation, a critical clue to solving bacterial drug resistance.

For the broader applicability of nanoscale SECM to various biological/microbiological systems, we recently demonstrated an ion-selective amperometric/voltammetric nanopipet electrode based on facilitated ion transfer (FIT) with an ion-selective ionophore, i.e., carbonate (${\text{CO}}_{3}^{2-}$) selective amperometric/voltammetric nanoprobes [46]. This electrochemical study, for the first time, revealed critical factors to govern nanoscale ${\text{CO}}_{3}^{2-}$-selective electrodes using broadly available Simon-type ionophores forming a covalent bond with ${\text{CO}}_{3}^{2-}$, such as slow dissolution of lipophilic ionophores in 1,2-DCE organic phase, activation for hydrated ionophores, peculiar solubility of hydrated ion–ionophore complex near the interface, and cleanness at the nanoscale interface, thereby achieving reproducible performance of nanoscale-ISE. These factors were experimentally confirmed by nanopipet voltammetry, where facilitated ${\text{CO}}_{3}^{2-}$ transfer was studied with a nanopipet filled with organic phase containing a trifluoroacetophenone derivative as ${\text{CO}}_{3}^{2-}$ ionophore VII by voltammetric and amperometric sensing of ${\text{CO}}_{3}^{2-}$ in water (Fig. 6C). Notably, reproducible voltammetric responses enabled us to explicitly evaluate the mechanism of FIT, where the dynamics of complexation between ${\text{CO}}_{3}^{2-}$ and ionophore VII follows the one-step electrochemical (E) mechanism controlled by both water-finger formation/dissociation and ion-ionophore complexation/dissociation in a concerted manner at the interface (Fig. 6D). Amperometric ${\text{CO}}_{3}^{2-}$-selective nanopipets were successfully applied to measure a ${\text{CO}}_{3}^{2-}$ concentration produced by metal-reducing bacteria, *Shewanella oneidensis* MR-1, as a result of organic fuel oxidation in bacterial growth media. Therefore, amperometric nano-${\text{CO}}_{3}^{2-}$ ISEs will be useful as nanoscale SECM probes to study microbial respiration and electrosynthesis by metal-reducing bacteria at a single cell level.

## 6. Acetylcholine-selective amperometry at neurons

Applications of nanoscale ion-selective amperometry to biological systems were demonstrated for the first time by the Shen group, who detected acetylcholine (ACh) at single live neurons [47,48]. The electrochemical detection of ACh is a highly demanded but challenging task because this important neurotransmitter is redox-inactive. An ion-selective electrode does not require the redox reaction of an analyte ion and was reported previously for both voltammetric and potentiometric detection of ACh without in-vitro or in-vivo applications to biological systems [140,141]. Specifically, an amperometric ACh-selective nanopipt was employed as an SECM tip to sensitively probe the in-vitro dynamic concentration of ACh released from living neuronal soma with high spatial and temporal resolutions [48]. The amperometric nanoelectrode was doped with dibenzo-18-crown-6 as an ionophore to demonstrate ACh selectivity against background ions in the cellular medium of the neuronal model, artificial seawater (ASW). The ACh selectivity was attributed to the binding of ACh to dibenzo-18-crown-6 as well as the relatively high hydrophobicity of ACh. The amperometric nanoelectrode was positioned at ~140 nm from the release sites of *Aplysia californica* by using SECM. An experimental approach curve was fitted with the simulated curve to determine not only the tip–neuron distance but also the permeability of the *Aplysia californica* neuron membrane to ASW ions within a range of 5 × 10^−2^ cm/s and 1 × 10^−1^ cm/s. Moreover, the amperometric tip detected the release of ACh in response to high-concentration K^+^ stimulation with a time resolution on the order of millisecond. The concentration of 2.7±1.0μM ACh released from the single cell was calculated from the maximum amperometric peak current measured *in situ*. The number of ACh molecules for each peak event was 80 (±20) × 10^−18^ mol, which corresponds to 4.8 (±1.3) × 10^7^ molecules.

**Fig. 7.**
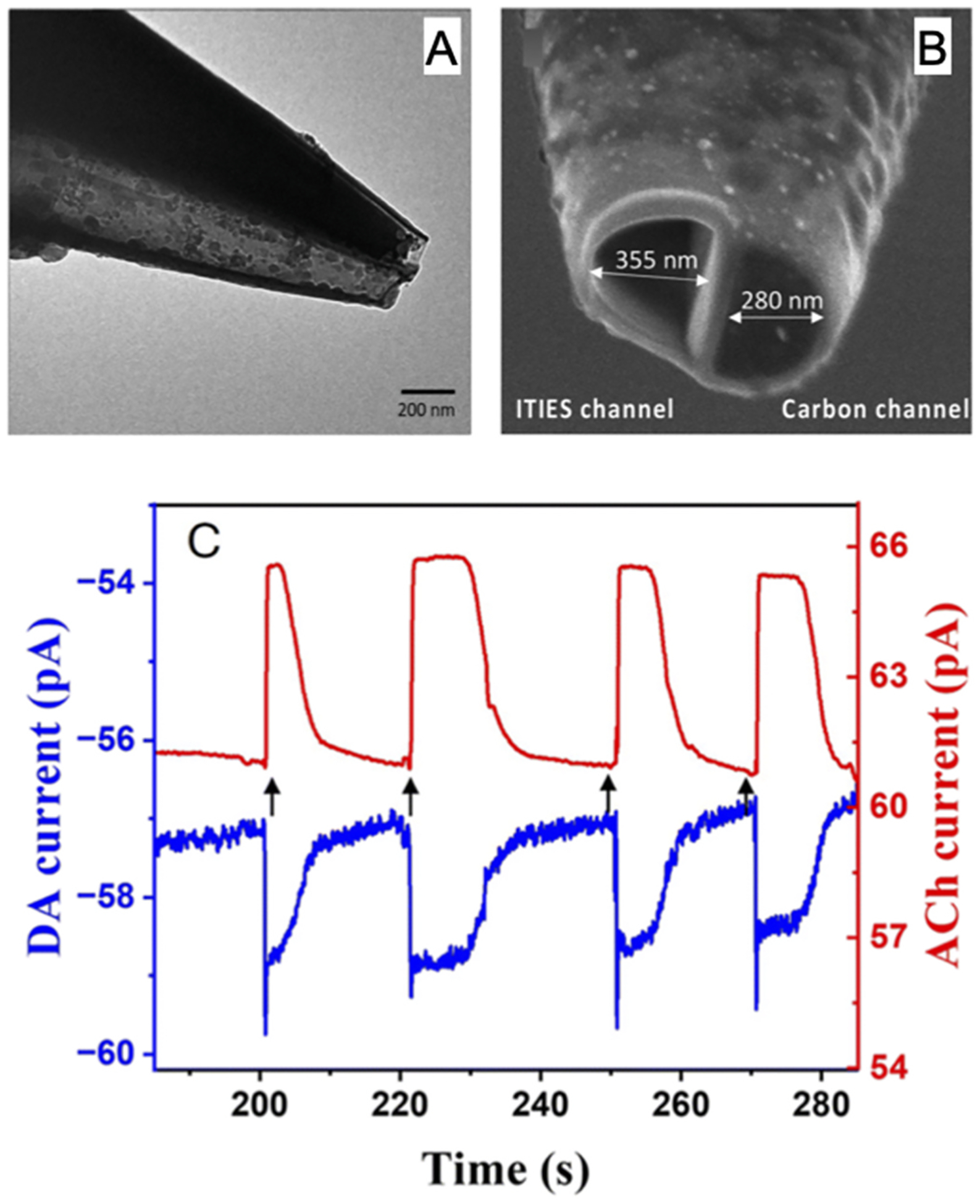
(A) TEM and (B) SEM images of the dual-channel nanopipet with carbon-deposited channel and FIB-milled open channel. (C) Amperometric responses of a dual-channel nanopipet to the injection of a mixture of dopamine (DA) and ACh as indicated by black arrows. The ACh channel was filled with the 1,2-DCE solution of 5 mM TDDATFAB. Reproduced from Ref. [142] with permission from the Royal Society of Chemistry.

Recently, the Shen group developed the dual-channel nanopipet electrodes to enable simultaneous amperometric detection of ACh and dopamine [142]. The novel electroanalytical method is crucial for understanding the interplay between the two neurotransmitters involved in various neurological events as well as relevant disorders and diseases [143]. A dual-channel nanopipet was prepared from a theta capillary by a laser-based puller to fill one channel with carbon for the oxidation of dopamine and the other channel with an organic electrolyte solution for the transfer of ACh. The advanced electrochemical nanosensors were fabricated and thoroughly characterized. Specifically, carbon was deposited by pyrolysis in one channel of the dual-channel nanopipet, which was milled by focused ion-beam technology to obtain a flat tip end. The single-channel deposition of carbon was confirmed by TEM (Fig. 7A) while the flat tip of the milled carbon-filled channel was confirmed by SEM (Fig. 7B). The open channel was silanized with N, N-dimethyltrimethylsilylamine and filled with the ionophore-free 1, 2-DCE solution of hydrophobic electrolytes, where a selective amperometric response was obtained for ACh with relatively high hydrophobicity. Each channel was characterized by steady-state voltammetry, which yielded the linear dependence of a diffusion-limited current response on either dopamine or ACh concentration as expected from eq (5). The potential of each channel was set for the diffusion-limited detection of the corresponding analyte to obtain simultaneous responses to both analytes (Fig. 7C).

**Fig. 8.**
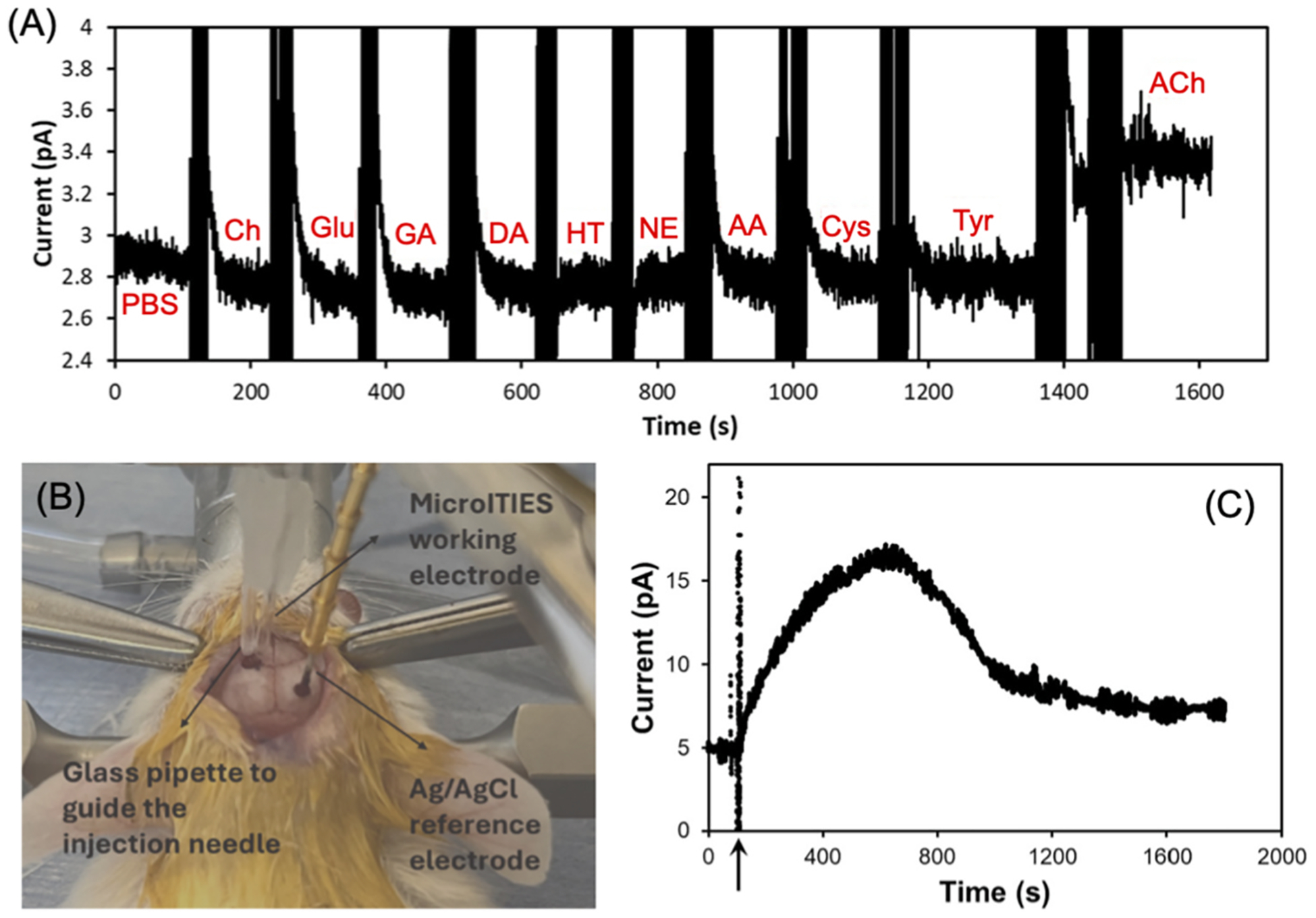
(A) In vitro amperometric responses of an ACh-selective micropipet to 50μM ACh and common interferents, i.e., choline (Ch), glutamate (Glu), gamma-aminobutyric acid (GA), dopamine (DA), serotonin (HT), norepinephrine (NE), ascorbic acid (AA), l-cysteine (Cys), and l-tyrosine (Tyr), in the phosphate-buffered saline (PBS). (B) A photo of the live mouse under the stereotaxic surgery with an amperometric ACh-selective microelectrode and an Ag/AgCl reference electrode as implanted in the brain. A glass pipet guides the injection needle near the micropipet electrode. (C) In vivo amperometric ACh response of an ACh-selective micropipet implanted in the cortex of a live mouse brain. Micropipets were filled with (A) and (C) the 2-nitrophenyl octyl ether solution of 0.1 M TDDATFAB and 5 mM heptakis(2,6-di-O-methyl)-β-cyclodextrin or (B) the 1,2-DCE solution of 5 mM TDDATFAB. Parts (A) and (C) were reproduced from Ref. [50] with permission from the American Chemical Society. Part (B) was reproduced from Ref. [49] with permission from Elsevier.

More recently, in vivo amperometry of ACh was enabled by using ion-selective micropipet electrodes [49,50]. The in-vivo electrochemical detection of ACh has been highly challenging owing to the intrinsic presence of choline as the interfering species produced by the hydrolysis of ACh. The selective in-vivo detection of ACh against choline, ascorbic acid, and other neurotransmitters was accomplished by using heptakis (2,6-di-O-methyl)-β-cyclodextrin [144] as an ACh ionophore in o-nitrophenyl octyl ether as the organic phase. The high ACh selectivity of the amperometric micropipet electrode with a tip diameter of 1–3μm was confirmed in vitro by both voltammetry and amperometry (Fig. 8A) [50]. A negligible current response was observed for several interferents including choline. An amperometric ACh-selective microelectrode was implanted in the cortex of a live mouse brain to detect ACh released from an injection needle (Fig. 8B) [49]. An in vivo current response to ACh was confirmed by injecting 2μL of 5 mM ACh solution into the brain as indicated by the noise of the microelectrode response around t=100 s after a stable background response was observed (Fig. 8C) [50]. The ACh solution was injected near the microelectrode to observe a gradual increase in the tip current. The tip current peaked at t=600 s and decreased gradually, which was not due to the loss of sensitivity because the electrode taken out from the brain demonstrated an amperometric ACh response to construct a post-surgery calibration curve. The calibration curve was used to convert the current response of the microelectrode to the ACh concentration. The concentration–time curve demonstrates the capability of the amperometric ion-selective microelectrode to quantitatively monitor the real-time ACh dynamics in vivo.

## 7. Perspectives

Remarkable progress has been made in the development and application of amperometric ion-selective nanoelectrodes to envision the future advancement of the already powerful method in selectivity and sensitivity. High selectivity is required for a wider range of applications to detect target ions in complex samples [145,146] including Cl^−^ [147] and K^+^ [148] in brains. Highly selective ionophores have been developed for potentiometry [10,149] and successfully applied to amperometry/voltammetry at micropipet electrodes [45,57,150] but to the nanoscale counterpart only recently [46]. Moreover, the highly selective detection of multiple ions is achieved by dynamic voltammetry [28,30, 31] in contrast to equilibrium potentiometry. Furthermore, a redox-inactive zwitterionic neurotransmitter, gamma-aminobutyric acid, was detected by amperometric ISEs [151,152]. A high sensitivity will enable us not only to detect lower concentrations of analyte ions but also to improve spatial resolution by employing a smaller nanopipet, which can be as small as ~10 nm [80]. The amperometric response of a smaller SECM tip is less affected by convection to achieve a steady state more quickly, thereby enabling faster imaging with higher temporal resolution [94]. Moreover, faster SECM imaging can be coupled with a more advanced algorithm than the standard constant-height imaging to simultaneously resolve the topography and reactivity of a complex substrate [153].

## Data Availability

No data was used for the research described in the article.
